# Rural Indonesian adolescents’ smoking behaviours during the COVID-19 pandemic: rapid survey and cotinine test of school-attend adolescents in Gunung Kidul, Yogyakarta

**DOI:** 10.1038/s41598-023-50123-2

**Published:** 2024-01-26

**Authors:** Fitriana Murriya Ekawati, Dhiana Ayu Novitasari, Dwi Astuti Dharma Putri, Novi Fitriyani, Zulfikar Ihyauddin

**Affiliations:** 1 Department of Family and Community Medicine, Faculty of Medicine, Public Health and Nursing, Universitas Gadjah Mada, Gedung Radioputro Lt 1. Sayap Barat, Jalan Farmako Sekip Utara Sleman, Yogyakarta, 55281 Indonesia; 2 Faculty of Medicine, Public Health and Nursing, Center for Child Health-Paediatric Research Office, Universitas Gadjah Mada, Yogyakarta, Indonesia; 3 Kartika Clinic 0729, Bantul Regency, Yogyakarta, Indonesia; 4 Paediatric Specialist Program, Faculty of Medicine, Public Health and Nursing, Universitas Gadjah Mada, Yogyakarta, Indonesia

**Keywords:** Medical research, Health care, Health services, Paediatrics, Public health

## Abstract

The COVID-19 pandemic is predicted to affect adolescent smoking behaviours. We aim to map profiles of adolescents’ smoking behaviours in a rural district in Indonesia during the COVID-19 pandemic and validate their smoking exposures using cotinine tests. This study applied an online survey followed by cotinine tests for high-school students in Gunung Kidul, Yogyakarta. The participants were asked to complete the survey and participate in a cotinine test. Univariate and multivariate regressions were performed to seek potential determinants of the smoking status and diagnostic accuracy of the cotinine test. A total of 281 participants completed the survey, with 19.6% (n = 55) and 22.8% (n = 64) being ever-smokers and current smokers. The impacts of the pandemics on their smoking behaviours were found in the urgency and numbers of daily smoked cigarettes. Univariate regression analysis revealed age, gender, learning mode, and whether father/friend smokes correlate with the adolescents’ smoking behaviours. Multivariate regression analysis revealed that the odds of planning to stop smoking were 0.01 (95% CI 0.001–0.22, p-value 0.003) for having positive attitudes towards cigarettes compared to none. Of the 65 cotinine tests, 19 tested positive, with the sensitivity and specificity of the cotinine test at 94.7% and 95.6%. The prevalence of adolescent smoking during the COVID-19 pandemic in Gunung Kidul is high, with the impacts of the pandemic on the urgency and number of cigarette smoke. There are opportunities to help them stop smoking by providing reliable quit-tobacco access and advocacy in collaboration with schools, parents, and health providers.

## Introduction

Up to a third of Indonesian men are smokers, and the prevalence has continued to increase in adults and adolescents in the last two decades. Recent surveys report that around 19.6% of Indonesian adolescents smoke and up to 85% of Indonesian adolescents have been exposed to tobacco^1,2^. This situation is alarming as the adolescent smoking status may initiate their smoking addiction in the future^3,4^, and may impact on their health and economic vulnerability^5^.

There are three large datasets so far available in Indonesia to provide initial data on the smoking behaviours of adolescents: the Indonesia Basic Health Research (BHS)^2^, Global Youth Tobacco Survey (GYTS)^6^, and Global School-based Student Health Survey (GSHS)^7^. These datasets report that the rate of adolescent smoking in Indonesia is very high, from 11.5 to 20.2% of the student population, and the main determinants for their smoking behaviours are their social interaction with smokers, easy access to tobacco products and tobacco advertisements.

Meanwhile, in the recent 3 years, the COVID-19 pandemic has significantly disrupted people’s social activities, including adolescents. Often, schools and social meetings shifted to teleconferences or online tasks. With these shifting mechanisms, adolescents cannot interact with their peers or easily access the tobacco kiosk close to their school. These situations may impact their adaptation and development, raising their struggle to adapt to the loneliness, which may lead to mental health issues, including their addictive behaviours toward substances and tobacco products^8,9^.

Unfortunately, with the changing situation during the pandemic, limited investigations are available to explore the rate of smoking behaviours among rural Indonesian adolescents. A recent survey in Jakarta reported an increased level of drinking but a reduced smoking rate among Indonesian adolescents during the pandemic^10^, while other research shows that their smoking behaviours were correlated with their education and stress level^11^. The smoking behaviours also correlate with their economic status and psychosocial support during the pandemic^9,12^. While there is a need to provide equal strategies to help students in rural areas change their behaviour, which is expected to invest in their future health^13,14^, however, previous studies on the substance abuses were predominantly conducted in the metropolitan cities. Limited investigation is available to evaluate whether the pandemic affected adolescents’ smoking behaviours in rural Indonesia, who have different backgrounds and social environments. Also, limited investigations are available to validate the adolescents’ smoking behaviours with their cotinine test as the gold standard of their cotinine exposures^15,16^. Therefore, this study has two aims to fill in the gap in the literature above: to describe profiles of adolescent smoking behaviours in rural areas in Indonesia during the COVID-19 pandemic and to validate their smoking exposures using cotinine tests. We also looked at the determinants of smoking behaviours and predictors of their intention to stop smoking.

## Materials and methods

### Design

The design of this study was a cross-sectional study using an online survey for high school students in grades X-XII (average age: 15–17 years old) followed by a voluntary cotinine test in Gunung Kidul, Yogyakarta province. Gunung Kidul is a district located in rural areas in Yogyakarta with limited access to the city and has 25 senior high schools with approximately ten classes per grade and 30–40 students in each class^17^. Of the number of high schools in the district, this study involved two schools in allowing adequate exploration of the smoking prevalence and the determinants of adolescent smoking behaviours in the district area^18^.

### Study settings

The setting of this study was in Yogyakarta province, Indonesia, of which 23.5% of its population (approximately 101,000 population) was smoking in 2018^2^. Based on the 2013–2018 Indonesian BHS survey, Yogyakarta was also in the top 15 highest smoking rate in Indonesia, and Gunung Kidul district has the highest level of adolescent smoking behaviour among other districts (Kota Yogyakarta, Sleman, Bantul and Kulon Progo)^2^. During the COVID-19 Pandemic, schools in Yogyakarta were conducted remotely since March 2020. The schools were also open and closed based on the condition in the region, i.e., the accumulative case number. If a region had no active case, the Indonesian government allowed its opening for students using strict health protocols^19–21^.

### Participants

This research was conducted in two vocational high schools in Gunung-Kidul district, Yogyakarta. The study inclusion criteria were adolescents attending grades X–XII, and the exclusion criteria were those currently with epilepsy/tuberculosis treatment that can interfere with the cotinine tests.

### Recruitment

Recruitment for the study was conducted in May–June 2021 using a convenient and snowballing sample design using the following procedures. Two vocational high schools in Gunung-Kidul were selected based on the authors’ professional networks of health providers. Representatives of the students’ council were also invited to assist in recruitment and data collection with permission from their parents. Then, they were asked to distribute the survey link to their schoolmates for the survey pages (informed consent forms included) to be filled in a month. In the following month, when the survey had ended, participants willing to participate in the cotinine test were invited to have the test in the school or be sent with the survey kits to their address.

### Data collection

The survey was conducted from June to July 2021. The survey pages consist of three parts. First is the informed consent page containing information about the study and consent from the prospective participants and parents. The second part is the demographic survey of the participants. The third part is the study questionnaires informed by the World Health Organisation’s GYTS survey in Bahasa Indonesia version^6^, and used questions to explore their activities and habits in the last 2 weeks before filling in the questionnaire (July–August 2021) to indicate their responses during the pandemic and minimise the recall bias. The online survey pages have also been validated with 15 validation participants, and the survey was conducted anonymously without asking for participants’ personal information. The English version of the questionnaire is attached in Supplementary File 1.

### Cotinine test device

At the end of the survey, participants were asked whether they were willing to participate in the cotinine test. The tests used rapid test cassette Cotinine (COT)^22^. It was conducted using the participant’s urine sample, which was accommodated in a jar and by dripping three drops of urine using a pipette into the litmus paper on the cotinine strip. The results would then appear within 15 s. One line on the kit indicates a positive cotinine result, which means smoking and two lines indicate a negative cotinine result or not smoking. The cut-off of this device is 200 ng/mL of cotinine in the urine^23^.

The cotinine test was conducted in school or self-tests by sending the kits via Post in July–August 2021. Participants who filled out the online survey were eligible to participate in the draw of 20 mobile plan vouchers IDR 50,000 (5 AUD), and those who participated in the cotinine test were given IDR 20,000 (2 AUD) as a token of their participation.

### Roles of the representatives of the student council and cadres

We anticipate that during the pandemic, students could not interact with their schoolmates regularly and with the external researchers, as such in the study. Explanations of the cotinine test also faced challenges for their acceptance at school due to concerns about the results being reported to the school. Therefore, we collaborated with the representatives of the students and the student council, who were recruited as cadres of *Posbindu (Pos Binaan Terpadu*/Eng: empowerment program to provide screenings for school communities), to ensure the important information related to the tests reached all students in each class^24^. The cadres were then trained once in each school to provide necessary information regarding the survey and the details of the cotinine test to their classmates. This information session was conducted in June 2021.

### Variables

The participants’ tobacco use was explored using the questions “During the past 30 days, how many days did you smoke cigarettes?” and “How old were you when you started to smoke?”. Based on the answers to those questions, we categorised the participants into three groups, i.e., current smokers for those who smoked cigarettes during the past 30 days; former smokers for those who ever smoked tobacco but did not smoke any during the past 30 days; and non-smokers for those who never tried to smoke tobacco.

### Data analysis

Descriptive statistics were used to describe the distribution of data on demographic characteristics (gender, education level, family economic level, parent’ education) and additional data from participants, including other risk factors (smokers in the inner circle, smoking history in the core family, and access to health care and cigarette). Univariate logistic regression analyses were performed for potential determinants of smoking, including age, sex, schooling method, parental working status, parental smoking status, close friends’ smoking status, positive attitude towards smoking, and negative attitudes towards smoking. Two following multivariable logistic regression analyses were also conducted to explore: (1) determinants of ever-planning to stop smoking during the pandemic among current smokers and (2) determinants of smoking during the pandemic. Both logistic regression analysis models were adjusted for potential confounders, including age, sex, schooling method, parental smoking status, close friends’ smoking status, positive attitude towards smoking, and negative attitudes towards smoking. Results from logistic regression analyses were expressed as odds ratio (OR) and adjusted odds ratio (aOR) along with the 95% confidence interval (95% CI). An interaction check for potential variables was performed before regression analysis, and no interaction was found. The goodness of fit test for the regression models was conducted using the Hosmer–Lemeshow test. Diagnostic accuracy from the self-reported questionnaire towards the results from the cotinine test as the reference test was explored using sensitivity, specificity, positive predictive value (PPV), and negative predictive value (NPV), while reliability was assessed using the kappa agreement test. All analyses were carried out using STATA version 14.2^25^.

### Ethics approval

This study obtained ethics approval from the Research Ethics Committee Faculty of Medicine, Public Health and Nursing, Universitas Gadjah Mada Number KE/FK/0322/EC/2021. This study has also obtained research permit approval from Gunung Kidul Local Health and Education Office. All methods were performed following the relevant guidelines and regulations, here ultimately based on the ethics guidance from the Research Ethics Committee Faculty of Medicine, Public Health and Nursing, Universitas Gadjah Mada.

### Consent to participate

Consent to participate was obtained from all of the participants in the study and/or their parents/legal guardian(s).

## Results

### Participants characteristics

A total of 375 participants attempted to fill in the survey, and 281 of them completed the online survey. The majority were male (n = 176, 62.6%), aged 15–16 years old (n = 149, 53%), having remote learning arrangement (online classes) (n = 256, 91.4%) and had one working parent (n = 162, 58.7%). Of the 281 participants, the proportion of current smokers (those who smoked tobacco in the past 30 days), former smokers (ever smoked tobacco but did not smoke in the past 30 days), and non-smokers (never smoked tobacco) were 22.8% (64/281), 19.6% (55/281), and 57.6% (162/281) respectively.

### Smoking background and habits

Of the 281 participants, most of their parents were primary (father) (n = 98, 36.8%) and (mother) high-school graduates (n = 93, 34.1%). As many as 210 participants (74.7%) had friends who smoke, 219 (78%) have been exposed to smoking or tobacco product, has close friends who are a smoker (n = 210, 74.7%), and expressed thoughts that smoking would make them more interesting to their peers (n = 89, 31.7%) (Table 1).

**Table 1:** Baseline characteristics of participants.

Variables	All samples n (%)	Ever-smokers n (%)	Current smokers n (%)	Non-smoker n (%)
Number of samples	281 (100)	55 (19.6)	64 (22.8)	162 (57.6)
Age				
≤ 14 years old	2 (0.7)	1 (1.8)	0 (0)	1 (0.6)
15–16 years old	149 (53.0)	30 (54.5)	22 (34.4)	97 (59.9)
≥ 17 years old	130 (46.3)	24 (43.7)	42 (65.6)	64 (39.5)
Sex				
Female	105 (37.4)	1 (1.8)	2 (3.1)	102 (63)
Male	176 (62.6)	54 (98.2)	62 (96.9)	60 (37)
Schooling method				
Offline class	5 (1.8)	0 (0)	4 (6.3)	1 (0.6)
Combination of online and offline	19 (6.8)	4 (7.3)	2 (3.1)	13 (8.1)
Online class	256 (91.4)	51 (92.7)	58 (90.6)	147 (91.3)
Parental working status				
Both parents do not work	21 (7.6)	5 (9.3)	0 (0)	16 (9.9)
One parent works	162 (58.7)	27 (50)	35 (57.4)	100 (62.1)
Both parents work	93 (33.7)	22 (40.7)	26 (42.6)	45 (28)
Father’s educational level				
Elementary school	98 (36.8)	16 (30.2)	19 (32.2)	63 (40.9)
Middle school	64 (24.1)	13 (24.5)	12 (20.3)	39 (25.3)
High school	89 (33.5)	21 (39.6)	24 (40.7)	44 (28.6)
College	15 (5.6)	3 (5.7)	4 (6.8)	8 (5.2)
Mother’s educational level				
Elementary school	92 (33.7)	17 (31.5)	20 (34.5)	55 (34.2)
Middle school	75 (27.5)	13 (24.1)	15 (25.9)	47 (29.2)
High school	93 (34.1)	19 (35.2)	22 (37.9)	52 (32.3)
College	13 (4.7)	5 (9.3)	1 (1.7)	7 (4.3)
Exposed to smokers at home or public places				
Not exposed	62 (22.0)	10 (18.2)	8 (12.5)	44 (27.2)
1–2 days	50 (17.8)	10 (18.2)	8 (12.5)	32 (19.7)
3–6 days	112 (39.9)	21 (38.2)	26 (40.6)	65 (40.1)
≥ 7 days	57 (20.3)	14 (25.4)	22 (34.4)	21 (13)
Parental smoking status				
Both parents do not smoke	113 (40.2)	20 (36.4)	18 (28.1)	75 (46.3)
Father smokes	161 (57.3)	34 (61.8)	42 (65.6)	34 (61.8)
Both parents smoke	2 (0.71)	0 (0)	2 (3.12)	0 (0)
Do not know	5 (1.78)	1 (1.82)	2 (3.12)	2 (1.23)
Close friends’ smoking status				
None smokes	64 (22.8)	7 (12.7)	3 (4.7)	54 (33.3)
Few smoke	210 (74.7)	46 (83.6)	56 (87.5)	108 (66.7)
All smoke	7 (2.49)	2 (3.6)	5 (7.8)	0 (0)
Exposure to anti-smoking campaign				
Yes	208 (74)	40 (72.7)	43 (67.2)	125 (77.2)
No/not sure	73 (26)	15 (27.3)	21 (32.8)	37 (22.8)
Learnt about the hazards of smoking at school				
Yes	140 (49.8)	25 (45.4)	36 (56.2)	79 (48.8)
No/not sure	141 (50.2)	30 (54.5)	28 (43.8)	83 (51.2)
Exposed to cigarette advertisement				
In electronic media	165 (58.7)	33 (60)	33 (51.6)	99 (61.1)
In kiosks/stores	180 (64.1)	39 (70.9)	43 (67.2)	98 (60.5)
Received stuffs from cigarette company	40 (14.2)	6 (10.9)	18 (28.1)	16 (9.9)
Free offer from cigarette sales	14 (5.0)	2 (3.6)	9 (14.1)	3 (1.9)
Positive attitude towards cigarettes				
I would have more friends	89 (31.7)	18 (32.7)	17 (26.6)	54 (33.3)
It would make them more interesting	26 (9.3)	3 (5.5)	12 (18.8)	11 (6.8)
It would make them more comfortable at a party/meeting	91 (32.4)	20 (36.4)	24 (37.5)	47 (29.0)
Negative attitude towards cigarettes				
Dangerous for health	267 (95.0)	53 (96.4)	55 (85.9)	159 (98.1)
Increasing risk of COVID-19 infection	93 (33.1)	15 (27.3)	15 (23.4)	63 (38.9)
Increasing risk of COVID severity	213 (75.8)	46 (83.6)	33 (51.6)	134 (82.7)

Among current smokers, 39 (62.9%) started to smoke at 12–15 and > 16 years (n = 17, 27.4%), and even a participant started to smoke at below seven years (n = 1, 1.6%). Most of them smoked 1–2 days in the last 30 days (n = 22, 36.1%), and 13 (21.3%) smoked most of the days. Tobacco products smoked are cigarettes (n = 58, 90.6%), and the average number of cigarettes smoked daily is 2–5 loosies (n = 23, 41.1%). Most participants purchased tobacco from kiosks or stores (n = 37, 62.7%) for any loosie (n = 28, 50%). Interestingly, most of them also planned to stop smoking (n = 47, 73.4%), had attempted to stop smoking (n = 43, 95.6%) and received advice to stop smoking during the pandemic (n = 43, 67.2%). The participants claimed that the pandemic had affected their smoking behaviours (n = 51, 79.7%), the number of cigarettes smoked (n = 12, 23.5%) and their urgency to smoke (n = 11, 21.6%) (Table 2).

**Table 2:** Characteristics of current smokers.

Variables	Current smoker n (%)
Age when smoking their first cigarettes	
< 7 years old	1 (1.6)
8–11 years old	5 (8.1)
12–15 years old	39 (62.9)
≥ 16 years old	17 (27.4)
Number of days smoking in the last 30 days	
1–2 days	22 (36.1)
3–5 days	7 (11.5)
≥ 6 days	19 (31.1)
Almost everyday (30 days)	13 (21.3)
Average number of cigarettes smoked per day	
1	19 (33.9)
2–5	23 (41.1)
6–10	11 (19.6)
≥ 11	3 (5.4)
Used other tobacco products beside cigarettes	
Yes	6 (9.4)
No	58 (90.6)
Ever planned to stop smoking during the pandemic	
Yes	47 (73.4)
No	17 (26.6)
Had attempted to stop smoking (among those who planned to stop smoking during the pandemic)	
Yes	43 (95.6)
No	2 (4.4)
Perception to be able to stop smoking when he/she attempted to	
Yes	54 (84.4)
No	10 (15.6)
Ever received advice to stop smoking during the pandemic	
Yes	43 (67.2)
No	21 (32.8)
Ways to get cigarettes	
Buy at stores/kiosks/vending machine	37 (62.7)
Received from other people	20 (33.9)
Others	2 (3.4)
Ways to buy cigarettes	
Buy in 1 pax	18 (32.1)
Buy in individual cigarette	28 (50.0)
Buy in 1 carton	9 (16.1)
Buy own tobacco	1 (1.8)
Plan on smoking in the next 12 months	
Would stop or probably stop smoking	9 (14.0)
Probably still smoking	49 (76.6)
Still smoking	6 (9.4)
Pandemic has affected smoking behaviour	
Yes	51 (79.7)
No	13 (20.3)
Effects were observed on	
Ways to smoke	3 (5.9)
Time to smoke	9 (17.6)
Friends who accompany when smoking	6 (11.8)
Number of cigarettes	12 (23.5)
Urgency to smoke	11 (21.6)
Need to stop smoking	10 (19.6)

### Determinants of smoking during the COVID-19 pandemic

The univariate regression analysis looking at the smoking determinants during the pandemic revealed that the participants’ age (15–16 years old) and gender (male) correlate with the participants smoking behaviours during the pandemic with OR 0.36 (95% CI 0.20–0.65, p-value 0.001), and OR 28.0 (95% CI 6.6–117.4, p-value < 0.001) respectively. Their behaviours also correlated with online learning OR 0.07 (95% CI 0.01–0.67, p-value 0.020) or hybrid OR 0.03 (95% CI 0.002–0.41 p-value 0.009), whether their father smokes OR 1.86 (95% CI 1.01–3.44, p-value 0.047), and whether their close friends are smokers (if few of them smoke OR 7.39 (95% CI 2.23–24.5, p-value 0.001) and all friends smoke OR 50.8 (95% CI 6.82–378, p < 0.001)). Having three positive beliefs toward cigarettes increases the odds of smoking (OR 0.38, 95% CI 1.21–12.3, p-value 0.022), while having two or more negative beliefs towards cigarettes decreased the odds of smoking, i.e., OR 0.15 (95% CI 0.31–0.71, p-value 0.017) and OR 0.11 (95% CI 0.02–0.56, p-value 0.008), respectively (Table 3).

**Table 3:** Potential determinants of smoking during the pandemic.

Determinants	Univariate analysis		Multivariate analysis^	
	OR (95% CI)	p-value	aOR (95% CI)	p-value
Age				
≤ 14 years old	–	–	–	–
15–16 years old	0.36 (0.20, 0.65)	0.001	0.49 (0.24, 1.02)	0.059
≥ 17 years old	1*	–*	1*	–*
Sex				
Female	–	–	–	–
Male	28.0 (6.6, 117.4)	< 0.001	33.9 (3.95, 292.6)	0.01
Schooling method				
Offline class	–	–	–	–
Combination of online and offline	0.03 (0.002, 0.41)	0.009	0.01 (0.0001, 0.4)	0.017
Online class	0.07 (0.01, 0.67)	0.020	0.03 (0.001, 1.00)	0.050
Parental working status				
Both parents do not work	–	–		
One parent works	0.71 (0.39, 1.27)	0.254		
Both parents work	1*	–*		
Parental smoking status				
Both parents do not smoke	–	–	–	–
Father smokes	1.86 (1.01, 3.44)	0.047	1.06 (0.47, 2.37)	0.878
Both parents smoke	1*	–	1*	–
Do not know	3.52 (0.54, 22.6)	0.185	0.42 (0.04, 3.92)	0.448
Close friends smoking status				
None smokes	–	–	–	–
Few smoke	7.39 (2.23, 24.5)	0.001	8.66 (1.49, 50.0)	0.016
All smoke	50.8 (6.82, 378)	< 0.001	29.4 (2.35, 367)	0.009
Positive attitude towards cigarettes**				
0	–	–	–	–
1	0.82 (0.43, 1.54)	0.540	0.61 (0.26, 1.40)	0.243
2	0.74 (0.28, 1.95)	0.541	0.36 (0.11, 1.20)	0.099
3	3.88 (1.21, 12.3)	0.022	3.25 (0.81, 13.1)	0.097
Negative attitude towards cigarettes^§^				
0	–	–	–	–
1	0.63 (0.13, 3.09)	0.575	1.25 (0.20, 7.77)	0.813
2	0.15 (0.31, 0.71)	0.017	0.33 (0.05, 2.09)	0.242
3	0.11 (0.02, 0.56)	0.008	0.22 (0.03, 1.53)	0.127

*Omitted because of collinearity.

**Positive attitudes towards cigarettes include (1) Would have more friends; (2) Would make he/she more interesting; (3) Would make he/she more comfortable in a party/meeting.

^§^Negative attitudes towards cigarettes include (1) Dangerous for health; (2) Increasing risk of covid infection; (3) Increasing risk of covid severity.

^Multivariate analysis showed a good fit of the data (p-value for goodness-of-fit test = 0.9997) adjust for covariates, including age, sex, schooling method, parental smoking status, close friends’ smoking status, positive attitude towards smoking, and negative attitudes towards smoking.

The results from the multivariable regression analysis showed that only a few variables had a statistically significant association with the students’ current smoking behaviour. The associations occurred on i.e., male gender (OR 33.9, 95% CI 3.95–292.6, p-value 0.01), the combination of online and offline schooling method (OR 0.01, 95% CI 0.0001–0.4, p-value 0.017), and when few and all friends smoke (OR 8.66, 95% CI 1.49–50, p-value 0.016 and OR 29.4, 95% CI 2.35–467, p-value 0.009, respectively).

### Predictors to stop smoking

Our univariate regression looking at the potential determinants of stopping smoking showed that only if current smokers had negative attitudes towards cigarettes, they have a higher potential to stop smoking (OR 20.5, 95% CI 2.81–149, p-value 0.003 for having two negative attitudes and OR 17.0, 95% CI 1.8–160, p-value 0.013 for having three negative attitudes). On the other hand, having all three positive attitudes towards cigarettes would decrease the odds of ever-planning to stop smoking (OR 0.05, 95% CI 0.008–0.26, p-value < 0.001).

These findings were consistent with the results from the multivariable logistic regression that having negative attitudes towards cigarettes would increase the odds of ever-planning to stop smoking (OR 22.1, 95% CI 1.36–359, p-value 0.029 for having two negative attitudes and OR 27.0, 95% CI 1.14–638, p-value 0.041 for having three negative attitudes). Meanwhile, after adjusting for covariates, the odds of planning to stop smoking were 0.01 (95% CI 0.001–0.22, p-value 0.003) for having three positive attitudes towards cigarettes compared to none (Table 4).

**Table 4:** Potential determinants of ever-planning to stop smoking during the pandemic.

Determinants	Univariate analysis		Multivariate analysis^	
	OR (95% CI)	p-value	aOR (95% CI)	p-value
Age				
≤ 14 years old	–	–	–	–
15–16 years old	1.30 (0.46, 3.66)	0.613	2.11 (0.50, 8.98)	0.309
≥ 17 years old	1*	–*	1*	–*
Sex				
Female	–	–		
Male	1*	–*		
Schooling method				
Offline class	–	–	–	–
Combination of online and offline	0.34	0.257	0.05 (0.002, 1.1)	0.058
Online class	1*	–*	1*	–*
Parental working status				
Both parents do not work	–	–		
One parent works	1.37 (0.48, 3.88)	0.552		
Both parents work	1*	–*		
Age when smoking their first cigarettes				
< 7 years old	–	–		
8–11 years old	1.38 (0.09, 19.6)	0.814		
12–15 years old	2.78 (0.23, 33.9)	0.424		
≥ 16 years old	1.87 (0.13, 26.3)	0.641		
Ever received advice to stop smoking during the pandemic				
No	–	–		
Yes	2.42 (0.86, 6.78)	0.093		
Parental smoking status				
Both parents do not smoke	–	–	–	–
Father smokes	0.69 (0.22, 2.13)	0.515	0.22 (0.02, 1.96)	0.177
Both parents smoke	1*	–*	1*	–*
Do not know	0.36 (0.03, 4.72)	0.434	0.099 (0.01, 70.9)	0.996
Close friends smoking status				
None smokes	–	–	–	–
Few smoke	2.28 (0.39, 13.0)	0.355	11.1 (0.43, 285)	0.145
All smoke	0.3 (0.03, 2.76)	0.288	0.42 (0.009, 20.9)	0.667
Positive attitude towards cigarettes**				
0	–	–	–	–
1	0.50 (0.12, 2.03)	0.335	0.46 (0.07, 2.92)	0.407
2	0.21 (0.04, 0.99)	0.05	0.13 (0.02, 1.01)	0.051
3	0.05 (0.008, 0.26)	< 0.001	0.01 (0.001, 0.22)	0.003
Negative attitude towards cigarettes^§^				
0	–	–	–	–
1	4.89 (0.76, 31.6)	0.096	1.92 (0.16, 22.8)	0.603
2	20.5 (2.81, 149.0)	0.003	22.1 (1.36, 359)	0.029
3	17 (1.80, 160.0)	0.013	27 (1.14, 638)	0.041

*Omitted because of collinearity.

**Positive attitudes towards cigarettes include (1) Would have more friends; (2) Would make he/she more interesting; (3) Would make he/her more comfortable in a party/meeting.

^§^Negative attitudes towards cigarettes include (1) Dangerous for health; (2) Increasing risk of COVID-19 infection; (3) Increasing risk of COVID severity.

^Multivariate analysis showed a good fit of the data (p-value for goodness-of-fit test = 0.2967) and adjusted for covariates, including age, schooling method, parental smoking status, close friends’ smoking status, positive attitude towards smoking, and negative attitudes towards smoking.

### Cotinine tests

Of the 281 participants who filled out the survey, 72 agreed to have a urine test, and 19 tested positive for consuming cotinine. Based on the survey questionnaire, one out of the 19 cotinine-positive participants admitted that he did not smoke but close friends and family who smoke. Meanwhile, three of the 53 participants with negative results admitted smoking.

Diagnostic accuracy test showed that 18 out of 20 current smokers based on questionnaire answers had positive urine cotinine, while 44 out of 45 non-smokers from questionnaire responses had negative results. This case led to sensitivity, specificity, positive predictive value (PPV), and negative predictive value (NPV) for the self-reported questionnaire of 94.7%, 95.6%, 90%, and 97.8%, respectively. The kappa agreement test showed high reliability, i.e., 0.89 (95% CI 0.77–1.0, p-value < 0.001).

## Discussion

Our survey has provided evidence of smoking behaviours of Indonesian rural high school-aged adolescents during the COVID-19 pandemic. The prevalence of adolescent smokers in this survey is high, with the dominance of male students, having online or hybrid online and offline classes during the pandemic and having friends or family members who are smokers. The pandemic impact their smoking behaviours on the number of cigarettes smoked and the urgency of smoking. While many perceived that smoking was bad for their health and included the risk of severe COVID-19 infection, only half received information to stop smoking during the pandemic. Unfortunately, the cotinine tests were only conducted in around a quarter of the total participants, with high predictive values after cross-checking the participants’ answers to the questionnaire.

Compared to the GYTS survey results^6^, our findings indicated a similar rate of adolescent tobacco smokers in Indonesia, with a moderate decreasing trend of adolescents who purchased tobacco at the kiosks or stores. The rate of adolescents wanting to stop smoking in our survey was also lower compared to GYTS; more are on those aged 15–16, and indicated that the adolescents would probably still smoke in the next 12 months^6^. Our results are also consistent with results of previous tobacco-related research in rural Indonesian adolescents, that their regions, family role, social pressure and image of masculinity affect their smoking behaviours^26–30^. Even in our survey, the impact of the perceived social pressure of smoking has outweighed their perceived knowledge that smoking is dangerous for their health^31,32^.

Our survey results are also significant as it seems the current adolescent smoker has a stronger smoking addiction than before the COVID-19 pandemic. This addiction was claimed as compensation for the increasing stress and anxiety level resulting from the pandemic, including the changing school from onsite to remote learning and their inability to connect face-to-face with peers^33^. The effects might also be worsened in disadvantaged adolescents, such as those living in rural areas, as in this study and those from low socioeconomic status. While the results of the cotinine tests in these high-school adolescents are also alarming but not surprising, given that tobacco exposure has also impacted toddlers and primary school students in Indonesia^34^. Unfortunately, with the high prevalence of smokers and the high proportion of male smokers and those in adolescence who smoke, Indonesia has not signed the World Health Organisation (WHO) Framework Convention on Tobacco Control (FCTC) due to its heavy pressure on the economic side of the country. However, this has attracted criticism that the income the tobacco industry has produced is not balanced with the health risks and expenditures of the population due to the impact of smoking^35^. Therefore, this research also aims to urge the government to reconsider their decision to approve the tobacco control convention.

Due to its high priority and impacts of smoking on adolescents and its significant health risks for them in the future, further research should carefully develop a suitable service for adolescents to help them quit smoking and collaborate with parents and schools. As represented by the results in this study, adolescent smoking behaviour was highly correlated with social pressures due to mixed learning methods; however, their belief that smoking is dangerous for their health and may impact the severity of the COVID-19 symptoms. These findings can assist and educate them about the danger of smoking and create an image that it is great to socialise without using tobacco. The quit-smoking service should be accessible and flexible concerning the effect of the pandemic and the need to maintain their wellbeing and provide them with a friendly and reliable space if students want to stop smoking or reduce their addiction to particular substances^34^.

Our study is one of a few research in Indonesia to explore the pandemic’s effect on adolescents’ smoking behaviour, which has been completed with urine cotinine tests to validate their tobacco exposures. This research, however, also has some limitations. The sample size used in the study research is small and may not represent the condition of adolescents in all rural areas in Indonesia. Our sampling methods may also raise recruitment bias as it is only conducted in two high schools in Gunung Kidul Yogyakarta. While this study was conducted, Indonesia was at the peak of the Delta variant outbreak of the COVID-19 pandemic. Schools were closed and all activities were conducted online, including how information and the survey were distributed to the students. This limitation might impact the survey participation, even though it has previously been anticipated with the roles of the student cadres. Our data also only report the adolescent demographic background that potentially affects their smoking behaviours, and does not investigate the complex correlation between the variables. Therefore, the audience should interpret the results of this study appropriately.

## Conclusions

The prevalence of high-school adolescent smokers in Gunung Kidul district in Yogyakarta during the COVID-19 pandemic was high. The pandemic’s impacts are particularly on the urgency to smoke and the number of daily smoked cigarettes. Factors associated with adolescent smoking behaviours were particularly the social environment, such as their family or peers who are smokers, and also the changing learning modes from offline to online/hybrid mode. Results of the cotinine tests also confirm the students’ exposures to tobacco with a high sensitivity and specificity rate compared to their answers based on the questionnaire. Further research is needed to provide accessible and reliable stop-smoking services for adolescents, collaborating with schools, families and health providers.

### Supplementary Information

Supplementary Information.

## Data Availability

The raw data of the study are available from the corresponding author, the data can be shared upon approval of appropriate request.
