# Supplementary material for: Rural Indonesian adolescents’ smoking behaviours during the COVID-19 pandemic: rapid survey and cotinine test of school-attend adolescents in Gunung Kidul, Yogyakarta
Source: Sci Rep. 2024 Jan 26;14:2208. doi: 10.1038/s41598-023-50123-2 (PMC10817887; doi:10.1038/s41598-023-50123-2)
Supplement: Supplementary file 1 — Supplementary Information. [file 41598_2023_50123_MOESM1_ESM.docx]

**Supplementary File 1. Questionnaires used in the study**

**Inform Consent**

Before filling out the questionnaire, please take your time to read the information about this research carefully. The research consent sheet can be downloaded via the following link: Research information sheet.

If you have or want further explanation regarding this research, please ask your questions to the research team.

1. After reading the research agreement, did you participate in this research?

| O | Yes |
| --- | --- |
| O | Not |

1. Have you also obtained parental consent to participate in this research?

| O | Yes |
| --- | --- |
| O | Not |

1. At the end of this survey we will draw a draw for participants who have completed filling out the survey to get a credit voucher of 50 thousand rupiah, do you want to take part in this lottery?

| O | Yes |
| --- | --- |
| O | Not |

1. If yes, please enter your active mobile number

|  |
| --- |

**This page is about your background**

Thank you for your willingness to fill out this questionnaire, this first page contains questions about your background.

1. How old are you?

| O 11 years or less |
| --- |
| O 12 years old |
| O 13 years old |
| O 14 years |
| O 15 years |
| O 16 years |
| O 17 years or older |

1. What is your gender?

| O Man |
| --- |
| O Woman |

1. Are you currently attending school?

| O Yes |
| --- |
| O No |

1. What level did you attend school?

| O Elementary School |
| --- |
| O Middle School/Equivalent |
| O High School/Equivalent |
| O College |

1. What grade are you in now?

| O 1 |
| --- |
| O 2 |
| O 3 |
| O 4 |
| O 5 |
| O 6 |
| O Semester learning at university |

1. Do you currently attend school in a school building?

| O I don't go to school |
| --- |
| O yes |
| O i study fully from home (online) |
| O I follow the face-to-face program alternately |

**family background during the Covid Pandemic**

1. During this Covid 19 Pandemic, did both of your parents work?

| O | Yes, my father works |
| --- | --- |
| O | Yes, my mother works |
| O | Yes, both my parents work |
| O | My parents don't work |
| O | I do not know |

1. What was your father's last education/stepfather/guardian?

| O | Elementary School |
| --- | --- |
| O | JUNIOR HIGH SCHOOL |
| O | SENIOR HIGH SCHOOL |
| O | College |
| O | I do not know |

1. What was the last education of your biological mother/stepmother/guardian?

| O | Elementary School |
| --- | --- |
| O | JUNIOR HIGH SCHOOL |
| O | SENIOR HIGH SCHOOL |
| O | College |
| O | I do not know |

1. What do you plan to do after completing your current education?

| O | Enter college |
| --- | --- |
| O | work |
| O | Marry |
| O | I do not know |

**Smoking Habits During the COVID-19 Pandemic**

1. How old were you when you first tried smoking?

| O | I never smoke |
| --- | --- |
| O | 7 years or younger |
| O | 8 or 9 years old |
| O | 10 or 11 years old |
| O | 12 or 13 years old |
| O | 14 or 15 years old |
| O | 16 years or older |

1. In the last 30 days, have you ever tried smoking even just one or two puffs?

| O | Yes |
| --- | --- |
| O | No |

1. In the last 30 days, how many days did you smoke?

| O | 0 days |
| --- | --- |
| O | 1 or 2 days |
| O | 3 to 5 days |
| O | 6 to 9 days |
| O | 10 to 19 days |
| O | 20 to 29 days |
| O | For a full 30 days |

1. In the last 30 days, how many cigarettes did you usually smoke per day?

| O | I have not smoked in the last 30 days (a month) |
| --- | --- |
| O | Less than 1 stick a day |
| O | 1 stick per day |
| O | 2 to 5 sticks per day |
| O | 6 to 10 sticks per day |
| O | 11 to 20 sticks per day |
| O | More than 20 sticks per day |

1. Have you tried any form of smoked tobacco products in the last 30 days other than cigarettes?

| O | Yes |
| --- | --- |
| O | No |

1. Do you use any form of smoked tobacco products other than cigarettes?

| O | Yes |
| --- | --- |
| O | No |

1. In the last 30 days, have you smoked or wanted to smoke in the morning?

| O | I do not smoke |
| --- | --- |
| O | No, I don't smoke or want to smoke in the morning |
| O | Yes, I smoke sometimes or want to smoke in the morning |
| O | Yes, I always smoke or want to smoke in the morning |

1. In the last 30 days, how soon after you smoked did you start to feel a strong urge to smoke again that is hard to ignore?

| O | I do not smoke |
| --- | --- |
| O | I never felt a strong urge to smoke again after smoking |
| O | In 60 minutes |
| O | 1 to 2 hours |
| O | More than 2 hours to 4 hours |
| O | More than 4 hours but less than a full day |
| O | 1 to 3 days |
| O | 4 days or more |

1. Have you ever tried any smokeless tobacco products (eg: chewed tobacco gum?)

| O | Yes |
| --- | --- |
| O | No |

1. In the last 30 days, have you used any form of smokeless tobacco products (eg: chewed gum?)

| O | Yes |
| --- | --- |
| O | No |

**Respondents' Attitudes to Quit Smoking**

1. Have you ever wanted to quit smoking in the last 30 days?

| O | I never smoke |
| --- | --- |
| O | Now I don't smoke anymore |
| O | Yes |
| O | No |

1. During the last 30 days, have you ever tried to quit smoking?

| O | I never smoke |
| --- | --- |
| O | Yes |
| O | No |

1. In the last 30 days, do you think you could quit smoking if you wanted to?

| O | I never smoke |
| --- | --- |
| O | I don't smoke Now |
| O | Yes |
| O | No |

1. During this Covid 19 pandemic, have you ever received advice/instructions on how to quit smoking? (choose one answer only)

| O | I never smoke |
| --- | --- |
| O | Yes, from a program or a health worker |
| O | Yes, from a friend |
| O | Yes, from a family member |
| O | Yes, from both, namely the program or health workers and friends or family members |
| O | I never take advice/hints |

1. Has this Covid 19 pandemic affected your smoking habit?

| O | I never smoke |
| --- | --- |
| O | I have stopped smoking |
| O | Yes |
| O | No |

1. If yes, at what point does smoking differ?

| O | Number of cigarettes |
| --- | --- |
| O | Smoking time |
| O | Friends when smoking |
| O | How to get cigarettes |
| O | Desire to smoke |
| O | Desire to quit smoking |
| O | There is no difference in smoking habits |

**Existence of Respondents as Passive Smokers**

1. In the last 30 days, on average, how many days a week were people smoking in your house, while you were at home?

| O | 0 days |
| --- | --- |
| O | 1 to 2 days |
| O | 3 to 4 days |
| O | 5 to 6 days |
| O | 7 days |

1. In the last 30 days, on average in a week how many days did someone smoke in front of you, in any closed public place, other than your home (such as in schools, shops, restaurants, shopping centers, cinemas)?

| O | 0 days |
| --- | --- |
| O | 1 to 2 days |
| O | 3 to 4 days |
| O | 5 to 6 days |
| O | 7 days |

1. Where do you get them (others) smoking in the room?

| O | School |
| --- | --- |
| O | House |
| O | Restaurant/place to eat |
| O | Market or shopping mall |
| O | Cinema |
| O | Worship place |

1. In the last 30 days, on average in a week how many days did someone smoke in front of you, in an outdoor public place (eg: playground, sidewalk, entrance to building, park, beach)?

| O | 0 days |
| --- | --- |
| O | 1 to 2 days |
| O | 3 to 4 days |
| O | 5 to 6 days |
| O | 7 days |

1. In the last 30 days, have you seen anyone smoking inside or outside the school building?

| O | Yes |
| --- | --- |
| O | No |

1. Does cigarette smoke from people who are smoking harm your health?

| O | Definitely not harmful to health |
| --- | --- |
| O | May not harm health |
| O | May be harmful to health |
| O | Definitely a health hazard |

1. Do you agree with the prohibition of smoking in closed public places (such as: schools, shops, restaurants, shopping centers, cinemas)?

| O | Yes |
| --- | --- |
| O | No |

1. Do you agree with the prohibition of smoking in open public places (such as: playgrounds, sidewalks, entrances to buildings, parks, beaches)?

| O | Yes |
| --- | --- |
| O | No |

**How Do Respondents Get Cigarettes?**

1. In the last 30 days, how did you get the cigarettes? (may choose more than one answer)

| O | I haven't smoked at all for the last 30 days |
| --- | --- |
| O | I bought it in the shop |
| O | I bought it from a street vendor |
| O | I bought it at the kiosk |
| O | I bought it from a vending machine |
| O | I got it from someone else |
| O | I got it another way |

1. In the last 30 days, has it ever happened that a cigarette dealer refused to sell you cigarettes because you were considered a minor?

| O | I have never tried to buy cigarettes in the last 30 days (a month) |
| --- | --- |
| O | Yes, someone once refused to sell me cigarettes because of my age |
| O | No, my age has never been a barrier to buying cigarettes |

1. During this Covid 19 pandemic or the last time you bought cigarettes, how did you buy them?

| O | I didn't buy cigarettes for the last 30 days |
| --- | --- |
| O | I bought 1 pack |
| O | I bought it retail |
| O | I bought it directly 1 carton |
| O | I bought it straight away 1 roll |
| O | I bought tobacco and rolled it myself |

**Smoking that you can get**

1. In the last 30 days, have you seen or heard any anti-smoking messages/calls in the media (such as: TV, radio, internet, billboards, posters, newspapers, magazines, cinemas)?

| O | Yes |
| --- | --- |
| O | No |

1. In the last 30 days, have you seen or heard any anti-smoking messages/calls at sporting events, concerts, gatherings?

| O | I haven't been to any sporting events, concerts, or get-togethers in the past month |
| --- | --- |
| O | Yes |
| O | Not |

1. In the last 30 days, have you seen any health warnings on cigarette packs?

| O | Yes, but I don't think much about it |
| --- | --- |
| O | Yes, and it made me think about staying away or quitting smoking |
| O | Not |

1. In this school year, did you learn about the dangers of smoking?

| O | Yes |
| --- | --- |
| O | Not |
| O | Don't Know For Sure |

**Advertising or promotion Tobacco (may include cigarettes, other smoked tobacco, and smokeless tobacco)**

1. In the last 30 days, have you seen people smoking on TV, videos or movies?

| O | I haven't seen TV, videos, movies in theaters in the last 30 days |
| --- | --- |
| O | Yes |
| O | Not |

1. In the last 30 days, have you seen advertisements and promotions of cigarettes in places of sale (such as: shops, kiosks)?

| O | I haven't visited the shop in the last 30 days |
| --- | --- |
| O | Yes |
| O | Not |

1. In the last 30 days, have you ever used an item that featured the name/image of a tobacco company or tobacco product such as: lighters, hats, clothes or glasses?

| O | Yes |
| --- | --- |
| O | Possible |
| O | No |

1. In the last 30 days, did you have any items such as: (t-shirts, bag pens) with a logo or brand of cigarette products on the item?

| O | Yes |
| --- | --- |
| O | No |

1. In the last 30 days, have you ever received an offer of free cigarettes from a cigarette salesperson?

| O | Yes |
| --- | --- |
| O | No |

**Your attitudes and beliefs about tobacco/cigarette use.**

1. If one of your best friends offered you a cigarette, would you smoke it?

| O | Definitely I won't suck it |
| --- | --- |
| O | Maybe I won't suck it |
| O | Maybe I'll suck it |
| O | Definitely I will suck it |

1. At any time, in the next 12 months, do you think you will smoke?

| O | Definitely not smoking |
| --- | --- |
| O | Probably won't smoke |
| O | Maybe will smoke |
| O | Will definitely smoke |

1. If someone has started smoking, do you think it will be difficult to stop smoking?

| O | It's definitely not hard to quit smoking |
| --- | --- |
| O | Maybe it's not that hard to quit smoking |
| O | It may be difficult to quit smoking |
| O | Hard to quit smoking |

1. Does smoking help make the smoker feel more comfortable or less comfortable at celebrations, parties, or other gatherings?

| O | More comfortable |
| --- | --- |
| O | Less comfortable |
| O | No effect |

1. Do you agree or disagree with the statement: “I think I might enjoy smoking cigarettes”

| O | I recently smoked a cigarette |
| --- | --- |
| O | Strongly agree |
| O | Agree |
| O | Don't agree |
| O | Strongly disagree |

**A smoking policy that may exist in your school.**

1. During the Covid 19 Pandemic, does your school have a general rule or policy that prohibits smoking for students inside the school building?

| O | Yes |
| --- | --- |
| O | Not |
| O | I do not know |

1. During the Covid 19 Pandemic, did your school issue a special policy that prohibits smoking for students outside the school building?

| O | Yes |
| --- | --- |
| O | Not |
| O | I do not know |

1. During the Covid 19 Pandemic, did your school issue a special policy that prohibits smoking for employees in school buildings?

| O | Yes |
| --- | --- |
| O | Not |
| O | I do not know |

1. During the Covid 19 Pandemic, did your school issue a special policy that prohibits smoking for employees outside the school building?

| O | Yes |
| --- | --- |
| O | Not |
| O | I do not know |

1. How well does your school enforce its policy (or rules) on smoking among students?

| O | There are no rules/policies regarding smoking in the school environment |
| --- | --- |
| O | Very good |
| O | Pretty good |
| O | Not good |

1. How well does your school enforce its policy (or rules) on smoking among school staff?

| O | There are no rules/policies regarding smoking in the school environment |
| --- | --- |
| O | Very good |
| O | Pretty good |
| O | Not good |

**Smoking habits in your neighborhood**

1. Did your parents smoke?

| O | Not |
| --- | --- |
| O | Yes, both (father and mother) |
| O | only dad |
| O | only mother |
| O | I do not know |

1. Does your closest friend smoke?

| O | Not |
| --- | --- |
| O | Some of them smoke |
| O | Mostly smoke |
| O | All smoking |

1. How many students in your class smoke?

| O | Mostly smoke |
| --- | --- |
| O | Half smoke |
| O | Some of them smoke |
| O | Nobody smokes |

1. Do you think young people who smoke have more or fewer friends?

| O | More friends |
| --- | --- |
| O | Fewer friends |
| O | No different from non-smokers |

1. Do you think smoking makes young people look more or less attractive?

| O | More interesting |
| --- | --- |
| O | Less attractive |
| O | No different from non-smokers |

1. Do you think smoking is harmful to your health?

| O | Of course not dangerous |
| --- | --- |
| O | Maybe harmless |
| O | It might be dangerous |
| O | Obviously dangerous |

1. Do you know the effect of smoking habits on Corona virus infection?

| O | I don't know the relationship between smoking and Corona infection |
| --- | --- |
| O | I know a little about the relationship between smoking and Corona infection |
| O | I really know the relationship between smoking and Corona infection |

1. Do you think smoking can affect the severity of Corona virus infection?

| O | Certainly not |
| --- | --- |
| O | Probably not |
| O | Maybe yes |
| O | sure yes |

1. Do you think it is safe to smoke for only a year or two and stop after?

| O | Not safe |
| --- | --- |
| O | Maybe not safe |
| O | Maybe safe |
| O | Safe |

1. Did your family members talk to you about the dangers of smoking?

| O | Yes |
| --- | --- |
| O | Not |

1. During the past 12 months, have you read school textbooks or books on smoking and health?

| O | Yes |
| --- | --- |
| O | Not |
| O | I don't have school books or health books |

1. During the past 12 months, did you discuss in your class the reasons why people your age smoke?

| O | Yes |
| --- | --- |
| O | Not |
| O | Not sure |

1. During the past 12 months, have you learned anything in your class about the effects of smoking such as: it makes your teeth yellow, causes wrinkles, or makes you smell bad?

| O | Yes |
| --- | --- |
| O | Not |
| O | Not sure |

1. During school hours, how often do you see the teacher smoking inside the school building?

| O | Every day |
| --- | --- |
| O | Sometimes |
| O | Never |
| O | Don't know |

1. During school hours, how often do you see the teacher smoking outside the school building?

| O | Almost every day |
| --- | --- |
| O | Sometimes |
| O | Never |
| O | Don't know |

1. In your opinion, should the sale of tobacco products to minors be prohibited?

| O | Yes |
| --- | --- |
| O | Not |

1. Do you believe that tobacco companies are trying to persuade young people under the age of 18 to smoke?

| O | Yes |
| --- | --- |
| O | Not |

**Desire to eat in adolescent and adolescent smokers**

Please provide your answer in the following answer options: A scale of 1 (Never), 2 (Rarely), 3 (Sometimes), 4 (often), or 5 (Always), according to your habits.

1. When I crave a food, I know that I can't stop once I start eating it

| Never | Rarely | Sometimes | Often | Always |
| --- | --- | --- | --- | --- |
| □ | □ | □ | □ | □ |

1. If I eat the food I crave, I often can't control myself and eat too much

| Never | Rarely | Sometimes | Often | Always |
| --- | --- | --- | --- | --- |
| □ | □ | □ | □ | □ |

1. Food cravings always make me think of ways to get what I want to eat

| Never | Rarely | Sometimes | Often | Always |
| --- | --- | --- | --- | --- |
| □ | □ | □ | □ | □ |

1. I feel that food is always on my mind

| Never | Rarely | Sometimes | Often | Always |
| --- | --- | --- | --- | --- |
| □ | □ | □ | □ | □ |

1. I feel that I am addicted to food

| Never | Rarely | Sometimes | Often | Always |
| --- | --- | --- | --- | --- |
| □ | □ | □ | □ | □ |

1. Every time I have a craving, I will make a plan to eat.

| Never | Rarely | Sometimes | Often | Always |
| --- | --- | --- | --- | --- |
| □ | □ | □ | □ | □ |

1. I crave food when I'm bored, angry, or sad

| Never | Rarely | Sometimes | Often | Always |
| --- | --- | --- | --- | --- |
| □ | □ | □ | □ | □ |

1. I can't control my appetite

| Never | Rarely | Sometimes | Often | Always |
| --- | --- | --- | --- | --- |
| □ | □ | □ | □ | □ |

1. When I start eating, I have a hard time stopping

| Never | Rarely | Sometimes | Often | Always |
| --- | --- | --- | --- | --- |
| □ | □ | □ | □ | □ |

1. I can't stop thinking about eating even though I try not to think about it

| Never | Rarely | Sometimes | Often | Always |
| --- | --- | --- | --- | --- |
| □ | □ | □ | □ | □ |

1. If I give in to certain food cravings, I lose all control.

| Never | Rarely | Sometimes | Often | Always |
| --- | --- | --- | --- | --- |
| □ | □ | □ | □ | □ |

1. Every time I crave food, I will keep thinking about it until I actually eat the food

| Never | Rarely | Sometimes | Often | Always |
| --- | --- | --- | --- | --- |
| □ | □ | □ | □ | □ |

1. If I crave a food, I am consumed by the thought of eating it

| Never | Rarely | Sometimes | Often | Always |
| --- | --- | --- | --- | --- |
| □ | □ | □ | □ | □ |

1. My emotions often make me want to eat

| Never | Rarely | Sometimes | Often | Always |
| --- | --- | --- | --- | --- |
| □ | □ | □ | □ | □ |

1. When there is an appetizing food near me, I find it hard to resist the temptation to eat it.

| Never | Rarely | Sometimes | Often | Always |
| --- | --- | --- | --- | --- |
| □ | □ | □ | □ | □ |

After completing this questionnaire, several participants will be randomly selected to perform a urine/urine test to measure cotinine levels at the nearest Puskesmas. This inspection process is not dangerous and if you are willing to take part in this inspection, you will get a transportation replacement fee of 20 thousand rupiah.

Are you interested in taking this examination?

1. Are you willing to take a urine sample examination at the nearest Puskesmas?

| O | Yes |
| --- | --- |
| O | Not |

1. Please include an active cellphone/telephone/WA number that we can contact.

|  |
| --- |
